# Eye and head movements while encoding and recognizing panoramic scenes in virtual reality

**DOI:** 10.1371/journal.pone.0282030

**Published:** 2023-02-17

**Authors:** Walter F. Bischof, Nicola C. Anderson, Alan Kingstone

**Affiliations:** Department of Psychology, University of British Columbia, Vancouver, BC, Canada; University of Lincoln, UNITED KINGDOM

## Abstract

One approach to studying the recognition of scenes and objects relies on the comparison of eye movement patterns during encoding and recognition. Past studies typically analyzed the perception of flat stimuli of limited extent presented on a computer monitor that did not require head movements. In contrast, participants in the present study saw omnidirectional panoramic scenes through an immersive 3D virtual reality viewer, and they could move their head freely to inspect different parts of the visual scenes. This allowed us to examine how unconstrained observers use their head and eyes to encode and recognize visual scenes. By studying head and eye movement within a fully immersive environment, and applying cross-recurrence analysis, we found that eye movements are strongly influenced by the content of the visual environment, as are head movements—though to a much lesser degree. Moreover, we found that the head and eyes are linked, with the head supporting, and by and large mirroring the movements of the eyes, consistent with the notion that the head operates to support the acquisition of visual information by the eyes.

## Introduction

People experience the visual world with their eyes, so it is only natural that visual researchers have been concerned with the role that the eyes play in human vision [1–5]. What has received relatively little attention, however, is the role that the head plays in delivering visual information to the eyes (although there are notable exceptions [6–10]. This omission is noteworthy because in everyday life it seems that we routinely move our head as well as our eyes to look at things in our environment [11–14]. For example, when people walk about their environment they normally turn their head to look at things that interest them, the result being that their eyes tend to remain relatively central with respect to the head [15]. Indeed, a recent paper by Foulsham and Kingstone [16] discovered that when modelling where people are looking, the best predictor is where the centre of the head is directed.

This combined movement of the head and eyes can be contrasted with how vision researchers routinely study human vision. In the lab the head is normally immobilized—either through instruction or by physically restraining the head—so that the only way an observer can view different regions of a visual scene is by moving their eyes. Historically this has been an understandable approach given past limitations of research software and equipment, which made eye tracking in a head free environmemt extremely challenging. Similarly, if one is focused on basic visual processes where head movements would be outside of the focus of investigation, then ensuring that they do not emerge is most reasonable. Nevertheless, given the natural tendency to move the head as well as the eyes outside of the lab, the exclusion of head movements in the lab risks overlooking the role that the head plays in vision and overstating the relative importance of the eyes.

There are cognitive consequences too. For instance, when observers are free to rotate their head in line with rotated text, they do so, to offset the cognitive load of mentally rotating the text [17]. Additionally there is a close link between head movements and human cognition, including memory [18–20]. For example, Solman and Kingstone [21] demonstrated that memory improves for items that are looked at when eye movements are accompanied by a head movement, presumably because it takes more energy to move the head and the eyes than the eyes alone (i.e., having worked harder to look at something people encode it more deeply into memory [22]).

A follow-up investigation by Solman et al. [23] examined if the principles of information acquisition were the same for the eyes and the head. This was done by yoking a horizontally- or vertically-oriented rectangular window to an observer’s eye or head movements. Eye movements were found to move in the direction of the visible information—horizontally when the window was oriented horizontally, and vertically when the window was vertical. Critically, head movements showed precisely the opposite and complementary pattern, moving vertically with a horizontal window, and horizontally with a vertical slot. Solman et al. concluded that these results are consistent with a nested system of effectors whereby the head favours exploration of unknown regions of visual space which are in turn exploited by the eyes.

Although these findings elucidate the relation of head and eyes in the exploration of scenes, there are still many basic unknowns: How do the head and eyes move when there is no gaze- or head-contingent window? Is memory performance predicted by both head movements and eye movements? And what role does the visual world itself, or an individual’s idiosyncratic movement tendencies [24], play in the way the head and eyes are moved to encode and recognize visual scenes? The aim of the present study was to address these fundamental questions. We did so by immersing participants in a 3D virtual reality (VR) environment through which they could view 360° panoramic scenes by moving their head and eyes freely to encode and recognize different aspects of these visual scenes. Such a design allows us to investigate how participants actively select different parts of the environment to explore and inspect using both their eyes and head during scene encoding and recognition [25–30]. We then assess the cognitive effect of head and eye movements on recognition performance, and the relative contribution of the participant versus the visual world on eye and head movement behavior using scan- and head-path comparisons.

## Materials and methods

### Participants

Twenty-one (6 male, 15 female; age range 18–22) undergraduate students from the University of British Columbia participated for course credit. All reported normal or corrected-to-normal vision. Participants provided informed consent prior to participation, and the study was approved by the ethics board of the University of British Columbia (H10-00527).

### Apparatus

The experiment was controlled by a desktop computer running an HTC Vive Virtual Reality device equipped with an SMI eye tracker. The HTC Vive headset has a resolution of 1080 x 1200 pixels, with a 110° by 113° field of view and a refresh rate of 90 Hz. The virtual experimental set-up was built using the Unity platform (Unity Technologies, 2017) [31]. The SMI eye tracker has a sampling rate of 250 Hz and was controlled using the SMI-designed Unity plugin. Tracking accuracy was maintained by performing a calibration every 20 trials. Calibration consisted of following a moving white circle with a red dot in the middle to 5 different regions. The SMI Unity plugin reports only success or failure of calibration, not accuracy, so calibration was repeated upon failure. Head movements (movements of the HTC Vive headset) were tracked using inertial measurement units inside the headsets that were calibrated via two infrared base stations (60 Hz) located on opposite corners of the room [32].

### Stimuli

**Fig 1:**
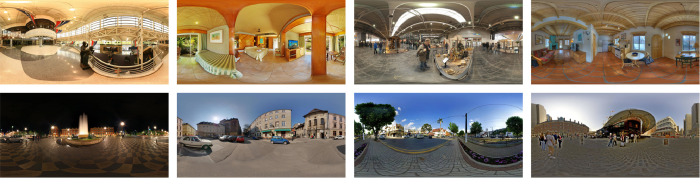
Examples of panoramic scenes. Examples of the panoramic scenes are shown that were used in the present experiment. Indoor scenes are shown in the top row, outdoor scenes in the bottom row.

The virtual space for both the encoding and recognition phases consisted of a sphere around the participant, onto which different omni-directional panoramic scenes were projected, effectively immersing the participant in the scenes. These scenes consisted of a mix of indoor and outdoor environments taken from the SUN360 Panorama Database [33], with a resolution of 4096 x 2048 pixels (Fig 1). The panoramas were chosen by selecting the first five in each of the categories of the database.

The panoramas were projected on the inside of the virtual sphere, with the participants’ head positioned in the middle. Binocular and motion depth cues were absent. The panoramas were defined in world coordinates, with longitudes in the range of −180° to 180° (from west to east) and latitudes in the range of −90° to 90° (from the south pole to the north pole). Further details are given below in the section ’General Spatial Analysis’.

### Procedure

Participants were instructed that there would be two phases to the study, an image encoding phase and an image recognition phase. In the encoding phase, participants were familiarized with the VR equipment and given the instructions to remember each of the scenes they were about to see for a later memory test. Each trial began with a uniform gray sphere with a fixation cross (at coordinates [0°, 0°] of the panoramas) that allowed us to monitor the tracker accuracy. After fixating on the cross, the participants pressed the ‘Spacebar’ key on a keyboard that they were holding to indicate that they were ready for the trial to begin. Each scene was presented for 10 seconds. There were 80 trials in the encoding phase (40 indoor and 40 outdoor scenes). This part of the experiment took approximately 20 minutes, after which participants were instructed to remove the headset and take a 5-minute break.

In the recognition phase, each trial began with a uniform gray sphere with a fixation cross. After fixating on the cross, participants pressed the ‘Spacebar’ key to indicate that they were ready for the trial to begin. Forty randomly chosen ‘old’ scenes from the initial set, and 40 ‘new’ scenes were presented for 10 seconds each. After each image was presented, instructions on a gray background indicated to participants to press the far left ‘Ctrl’ key if they had seen the scene before, and the right keypad ‘Enter’ key if they had not.

The participants were seated and held a keyboard that was connected by a long (3 m) cable. The starting position of the exploration was non-randomized, and to ensure that participants returned to the same position on each trial, they were asked to fixate the fixation cross before the next trial was presented.

### Data analysis

There is substantial confusion in the eye movement field regarding the definition of fixations and saccades, in particular when eye movements are combined with free head movements [34]. In our study, participants were situated in the centre of a virtual sphere and looked at the panoramic scenes with free head movements. We chose to define the measures below primarily with respect to the stimulus sphere, to allow expressing eye and head measures, and their comparison, with respect to a common coordinate frame. In this context, we now define terms related to the eyes and head. For a more detailed explanation of how to extract and use these and other VR eye and head measures see [35].

The eye direction vector was recorded using the binocluar direction vector at 250 Hz while the position of the headset and its orientation were recorded at a rate of approximately 70 Hz rate, with the exact timing being dependent on the Unity system [31]. The position and orientation of the headset were linearly interpolated to 250 Hz using spherical linear interpolation [36] of the headset quaternion.

The main eye analyses refer to the eye vector in a world coordinate frame, which is often referred to as gaze in the literature. It was computed by combining head direction with the eye direction (in head-defined coordinates). More precisely, the eye direction in world coordinates was computed by adding the eye direction vector to the headset quaternion [36]. An eye hit point, what we call an “eye point” for short, is defined as the intersection of the eye vector (in world coordinates) with the virtual sphere on which the panoramas are projected and is described by the sphere coordinates (longitude and latitude). In additional analyses, we also analyze the characteristics of the eye direction vector in head-defined coordinates.

With respect to the head, we define the head hit point, or “head point,” as the intersection of the vector pointing forward from the face with the virtual sphere, also defined by longitude and latitude of the panorama. Head movements with the VR viewer are usually very smooth and do not permit identifying stable points from the raw data. For this reason, we define a head fixation as the average head point during a fixation. Head shifts are defined as the angular differences between successive head fixations.

Fixations are defined as stable eye points on the sphere and are extracted from the unfiltered eye data using the Identification by Dispersion–Threshold algorithm adapted for use with spherical stimuli (IDT; [37, 38]), applying a minimum duration of 80 ms and a maximum dispersion threshold of 3° angular distance. Saccades are defined as angular differences between successive fixations.

In the spatial analyses, we also determined exploration tendencies. The exploration tendency of the eyes (in degrees visual angle) was calculated by taking the average of the great circle distance between each fixation position and the initial fixation position. Similarly, the exploration tendency of the head (in degrees visual angle) was calculated by taking the average of the great circle distance between each head fixation and the initial head fixation.

In the following sections, the data analyses focus on two aspects of the results, a purely spatial analysis and a spatio-temporal analysis. In the spatial analysis, the spatial distributions of eyes and head points are examined and compared directly, whereas the spatio-temporal analysis focuses on the cross-recurrence analysis of eye and head patterns.

## Results

### General spatial analysis

The panoramas were projected on the inside of the virtual sphere, with the participants’ head positioned in the middle. For the spatial analyses, panorama images and data were projected on a flat surface, an equirectangular (or equidistant) projection. This projection maps meridians into vertical straight lines of constant spacing, introducing strong distortions near the poles compared with the equator (the horizon area), with the panorama image stretched proportional to 1/cos(latitude). For this reason, data in the polar regions have to be interpreted carefully.

### Eyes: Spatial analysis of encoding and recognition

**Fig 2:**
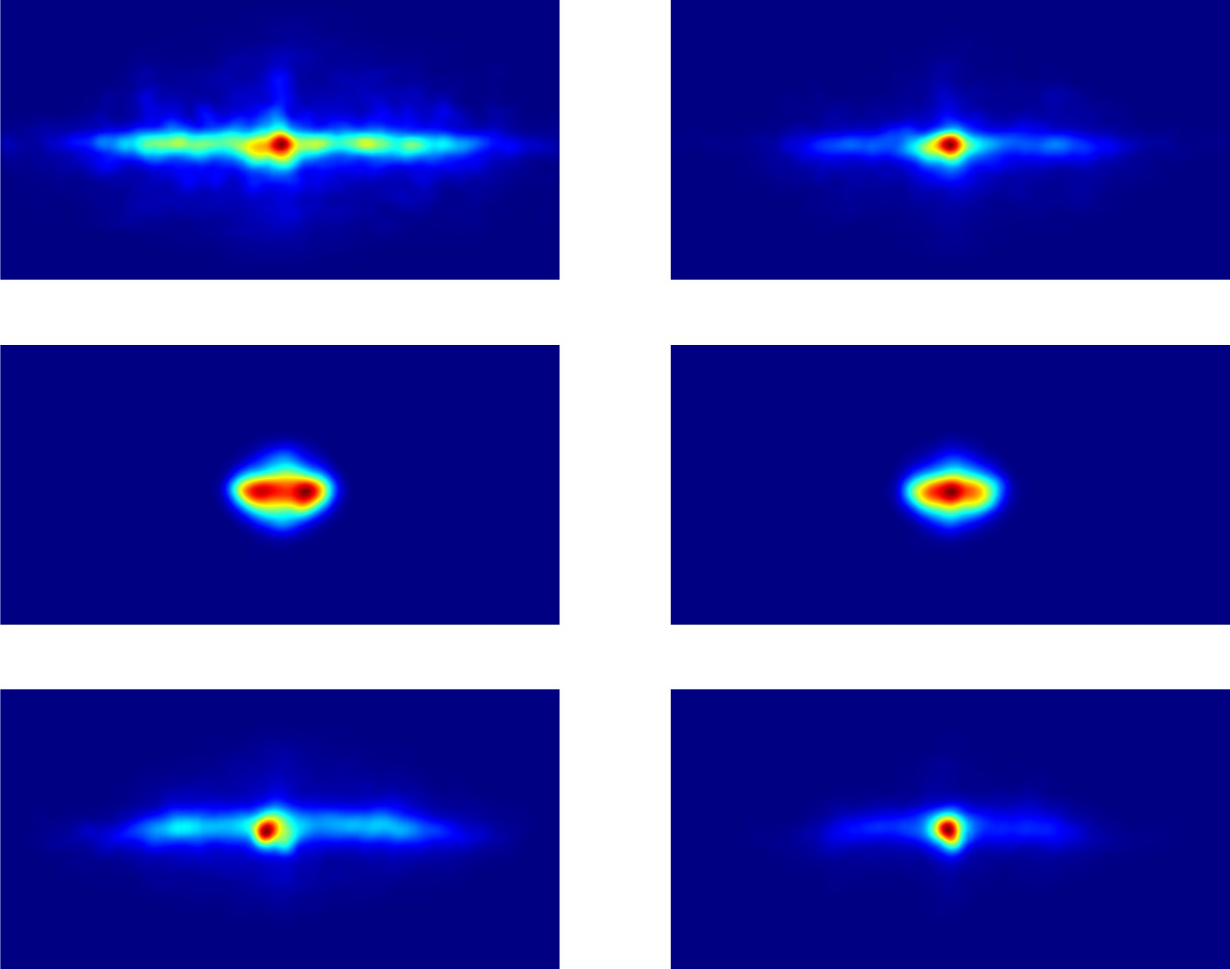
Encoding and recognition heatmaps. Encoding heatmaps are presented in the panels on the left side and recognition heatmaps in the panels on the right side. The top row shows the heatmaps of the eyes in world coordinates, the middle row the eyes in head-centered coordinates, and the bottom row the head positions in world coordinates. The coordinates of the heatmaps are all shown with longitudes in the range of −180° to 180° (from west to east) and latitudes in the range of −90° to 90° (from the south pole to the north pole). Note that for ease of comparison, the eyes in head-centered coordinates are shown with the same coordinates even though eyes in head coordinates are restricted to longitudes of about −70° to 70° (from west to east) and latitudes of about −60° to 60° (from south to north).

Generally, fixations are concentrated along the horizon (see Fig 2), a bias that has been previously found [27, 30]. Table 1 shows the fixation spreads, i.e., the median standard deviations of longitudes and latitudes for the encoding and recognition phases. The fixation spreads for longitudes are larger for the encoding phase than for the recognition phase, exact U-test, n_1_ = n_2_ = 42, p < 0.001. Similarly, the fixation spreads for latitudes are larger for the encoding phase than for the recognition phase, exact U-test, n_1_ = n_2_ = 42, p < 0.001. The fixation spreads during the encoding phase are larger for longitudes than for latitudes, exact U-test, n_1_ = n_2_ = 42, p < 0.001, and the fixation spreads during the recognition phase are also larger for longitudes than for latitudes, exact U-test, n_1_ = n_2_ = 42, p < 0.001.

**Table 1:** Spreads (median standard deviations) of eye positions.

	Longitude (deg)	Latitude (deg)
Encoding	67.33	21.41
Recognition	50.25	17.66

These results indicate that the spread of fixations was larger in the horizontal direction than in the vertical direction, consistent with previous work [27, 30, 39]. Furthermore, the spread of fixations was larger for the encoding phase than for the recognition phase. This suggests that during encoding participants focus on the exploration of the stimuli, whereas during recognition, they focus on a subset of the scene to check whether it has been seen before. To what extent, if any, having the same starting rotation contributes to this result is a question worthy of future investigation.

**Fig 3:**
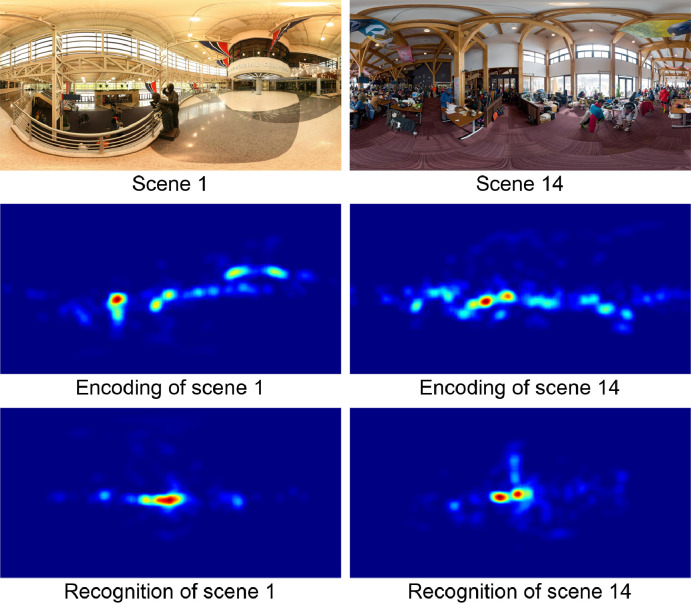
Fixation heatmaps for the encoding and recognition of two sample scenes.

A more detailed spatial comparison of encoding and recognition can be obtained by analyzing the fixation heatmaps. Fig 3 shows heatmaps for the encoding and recognition of two different images, with encoding heatmaps shown in the middle panels and recognition heatmaps (for the same scenes) shown in the bottom panels. The heatmaps were obtained from the fixation maps using a Gaussian filter with σ = 4° angular distance. Similarity between the encoding and recognition heatmaps should yield average correlations of the same scenes (e.g., between the encoding heatmap of scene 1 and the recognition heatmap of scene 1; see Fig 3) being higher than the average correlations between encoding and recognition heatmaps of different scenes (e.g., between the encoding heatmap of scene 1 and the recognition heatmap of scene 14; see Fig 3). A statistical analysis showed that average correlations between the encoding and recognition heatmaps for the same scenes ($r_{ER}^{\mathrm{same}}=0.814$) were significantly higher than the average correlations between encoding and recognition heatmaps for different scenes ($r_{ER}^{\mathrm{different}}=0.391$), t(39) = 19.98, p < 0.001. These correlations used latitude-dependent weights, as detailed in S1 Appendix.

The original images are shown in the top panels, the heatmaps of the encoding fixations in the middle panels, and the heatmaps of the recognition fixations in the bottom panels. The heatmaps are color-coded, with red indicating the strongest values and blue indicating the weakest values.

To further explore the characteristics of spatial eye movements, they were analyzed in head-centered coordinates rather than world coordinates. The heatmap of the eye positions in head-centered coordinates are shown in Fig 3, and the average standard deviations of longitudes and latitudes for the encoding and recognition phases are given in Table 2. The spreads for longitudes are similar for the encoding phase and the recognition phase, exact U-test, n_1_ = n_2_ = 42, p = 0.060; and spreads for latitudes are also similar for the encoding phase and the recognition phase, exact U-test, n_1_ = n_2_ = 42, p = 0.051. The spreads are larger for longitudes than for latitudes during the encoding phase, exact U-test, n_1_ = n_2_ = 42, p < 0.001, and during the recognition phase, exact U-test, n_1_ = n_2_ = 42, p < 0.001.

**Table 2:** Spreads (median standard deviations) of eye positions in head-centered coordinates.

	Longitude (deg)	Latitude (deg)
Encoding	16.22	10.59
Recognition	15.28	9.63

The spatial analysis of eye movements in world-coordinates yielded several results. First, the analysis showed a strong horizontal bias. Second, the spread of eye movements was larger in the encoding phase than in the recognition phase. This is consistent with the interpretation that in the encoding phase participants explored the scenes, and in the recognition phase they focused on one or more regions that matched their stored representations. Third, the correlational analysis of the encoding and recognition heatmaps indicates that people looked at the same regions of interest in the scenes during encoding and recognition.

### Head: Spatial analysis of encoding and recognition

An analysis of the head point patterns showed that they are also concentrated along the horizon of the scenes. Table 3 shows the average standard deviations of longitudes and latitudes for the encoding and recognition phases. The head spreads for longitudes are larger for the encoding phase than for the recognition phase, exact U-test, n_1_ = n_2_ = 42, p < 0.001. Similarly, the head spreads for latitudes are larger for the encoding phase than for the recognition phase, exact U-test, n_1_ = n_2_ = 42, p < 0.01. The head spreads during the encoding phase are larger for longitudes than for latitudes, exact U-test, n_1_ = n_2_ = 42, p < 0.001, and the head spreads during the recognition phase are also larger for longitudes than for latitudes, exact U-test, n_1_ = n_2_ = 42, p < 0.001.

**Table 3:** Spreads (median standard deviations) of head positions.

	Longitude (deg)	Latitude (deg)
Encoding	57.01	16.20
Recognition	39.90	11.95

A comparison of Tables 1 and 3 shows that head spread follows the same pattern as the eyes spread, but at a smaller absolute level. The statistical analysis shows that for both encoding and recognition, and for both longitudes and latitudes, the head spreads are smaller than the eye movement spreads, all exact U-tests, n_1_ = n_2_ = 42, p < 0.001.

**Fig 4:**
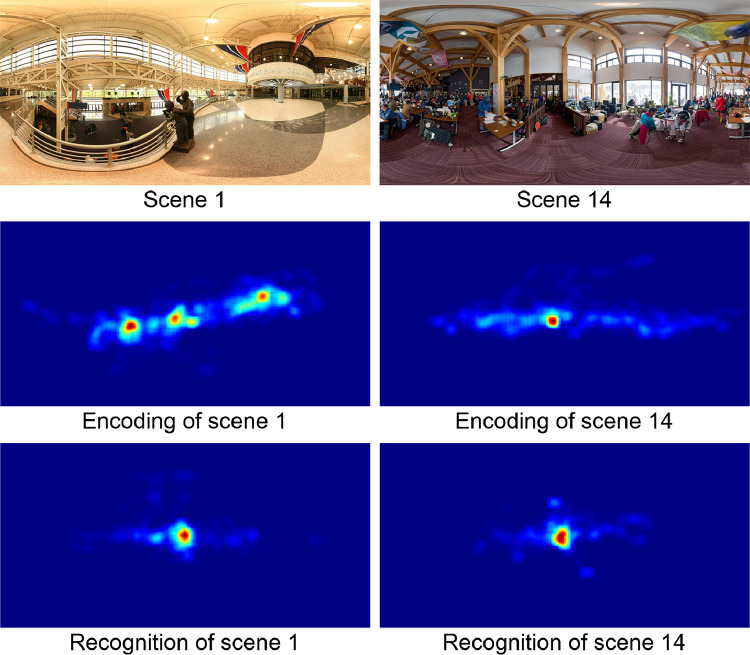
Head position heatmaps for two sample scenes. The original images are shown in the top panels, the heatmaps of the encoding head positions in the middle panels, and the heatmaps of the recognition head positions in the bottom panels. The heatmaps are color-coded, with red indicating the strongest values and blue indicating the weakest values.

A more detailed spatial comparison of encoding and recognition can be obtained by analyzing the head position heatmaps. Fig 4 shows head position heatmaps for the encoding and recognition of an indoor image on the left and the encoding and recognition of an outdoor image on the right. Encoding heatmaps are shown in the middle panel and recognition heatmaps (of the same scenes) in the bottom panels. The scenes are identical to those in Fig 3. The heatmaps were obtained from the head point (the location on the sphere were the head is pointing) maps using a gaussian filter with σ = 4° angular distance. Similarity between the encoding and recognition heatmaps should yield average correlations of the same scenes (e.g., between the encoding heatmap of scene 1 and the recognition heatmap of scene 1; see Fig 3) being higher than the average correlations between encoding and recognition heatmaps of different scenes (e.g. between the encoding heatmap of scene 1 and the recognition heatmap of scene 1; see Fig 4) should be higher than the average correlations between encoding and recognition heatmaps of different scenes (e.g. between the encoding heatmap of scene 1 and the recognition heatmap of scene 14; see Fig 4). A statistical analysis shows that the correlations between encoding and recognition heatmaps for same scenes ($r_{ER}^{s\mathrm{ame}}=0.934$) were significantly higher than the average correlations between heatmaps of different scenes ($r_{ER}^{\mathrm{different}}=0.722$), t(39) = 8.65, p < 0.001 (using latitude-dependent weights; see S1 Appendix).

To further explore the relation between eyes and head, the eye and head heatmaps of same scenes (e.g., eye heatmap of scene 1 and head heatmap of scene 1) were compared to the eye and head heatmaps of different scenes (e.g., eye heatmap of scene 1 and head heatmap of scene 3). The average heatmap correlations between eye and head for same scenes ($r_{GH}^{same}=0.625$) were significantly higher than the average correlations of different scenes ($r_{GH}^{different}=0.446$), t(39) = 8.29, p < 0.001. Similarly, in the recognition phase, the average heatmap correlations between eye and head for same scenes ($r_{GH}^{same}=0.684$) were also significantly higher than the average correlations of different scenes ($r_{GH}^{different}=0.575$), t(39) = 4.82, p < 0.001.

These results confirm the suggestion that eye and head followed similar movement patterns, both in the encoding phase and the recognition phase. These data converge with other studies [39, 40] showing that observers keep their eyes oriented within 15–20 degrees of the head-defined viewpoint. Given the robustness of this finding, one promising line for future studies will be to design investigations that are explicitly aimed at understanding the manner that the head responds to changes in eye-in-head coordinates, and how that relationship changes with different tasks and external environments (see also [41]).

### Eye and head movements and memory performance

In light of the results indicating that eye and head movements followed similar patterns during encoding and recognition phases, an important question is whether this overlap is functionally related to recognizing the scenes. To address this question we examined the extent to which various eye and head movement measures made during encoding and recognition predicted memory performance. Note that in general recognition accuracy was very good, with nearly 92% of all the scenes being recognized correctly. There are several potential reasons for the high recognition rate; first the large variation of scenes making the scenes almost uniquely identifiable and second, the stability of the starting positions by requring that the participants fixated the fixation cross at the beginning of each trial. It should be noted, however, that despite these considerations, we observed substantial head movements in the experiment.

Table 4 and the sections below present general eye movement measures, namely number of fixations per trial, mean fixation duration, exploration tendency of the eyes, and exploration tendency of the head. These measures are reported for the encoding phase, the recognition of ‘old’ images (which were presented in the encoding phase), and the recognition of ‘new’ images (which were not presented in the encoding phase). The eye movement measures are reported separately for correct and incorrect responses, as described in detail below.

**Table 4:** Means and standard deviations of general eye movement measures.

	Encoding	Old	New
Number of fixations per trial			
• Correct (Hit/Correct Rejection)	39.27 (3.39)	32.31 (8.91)	36.53 (6.82)
• Incorrect (Miss/False Alarm)	38.14 (5.09)	38.46 (6.65)	39.06 (6.83)
Mean fixation duration (ms)			
• Correct (Hit/Correct Rejection)	196.3 (19.3)	216.6 (24.1)	203.7 (18.0)
• Incorrect (Miss/False Alarm)	199.2 (31.7)	192.6 (17.4)	189.9 (19.1)
Exploration tendency of eyes (degrees)			
• Correct (Hit/Correct Rejection)	59.82 (10.46)	37.70 (15.9)	45.15 (15.2)
• Incorrect (Miss/False Alarm)	57.14 (12.02)	50.48 (15.6)	54.21(20.3)
Exploration tendency of head (degrees)			
• Correct (Hit/Correct Rejection)	49.72 (11.27)	30.40 (7.97)	36.75 (9.47)
• Incorrect (Miss/False Alarm)	47.35 (12.39)	42.35 (11.60)	44.75 (18.10)

For encoding, we report eye and head movement measures for only those images that were later included in the recognition phase, separately for those images that were later recognized correctly (Hits) and incorrectly (Misses). Note, however, that there were no differences in any of the eye and head movement measures between the images that were included in the recognition phase and those that were not (all t’s < 1.88, all p’s > 0.21). One subject was removed from the analysis of the encoding data for having a perfect memory performance score, while eight subjects were removed from the analysis of the recognition data for missing at least one category of responses (e.g., one subject made no False Alarms).

Below, we provide detailed analyses of the number of fixations, the mean fixation durations, and the exploration tendency of eyes and head. We fitted separate generalized linear mixed-effects models (GLMM [42]) for each measure to determine whether it predicted performance (on its own) over and above any variance accounted for by the random effects of image and participants. The analyses were done separately for encoding and recognition. By including participants and images as random effects, the GLMM’s take into account a given person’s ability to remember images, as well as a given image’s memorability. Likelihood ratio tests were conducted using the ‘mixed’ function from the afex package [43], and approximated p-values from Wald z-statistics were used for model estimates. For ease of interpretation, log odds output from the GLMM’s were converted to odds ratios using the sjPlot package [44].

### Number of fixations

In the encoding phase, there were a greater number of fixations in images that were correctly remembered (Hits), compared to those that were not (Misses), χ^2^(1) = 10.66, p = 0.001, Odds Ratio = 1.37, CI = [1.14, 1.65], z = 3.36, p < 0.001. Note that due to singularity issues arising from near-zero variance for the random effect of subject, it was dropped from the model, following [45]. In the recognition phase, there were significantly fewer fixations for old images that were correctly recognized (Hits) compared to those that were not (Misses), χ^2^(1) = 49.36, p < 0.001, Odds Ratio = 0.33, CI = [0.23, 0.47], z = -5.99, p < 0.001. Similarly, for new images, there were significantly fewer fixations for correct responses (Correct Rejections) compared to incorrect responses (False Alarms), χ^2^(1) = 5.22, p = 0.022, Odds Ratio = 0.60, CI = [0.38, 0.95], z = -2.16, p = 0.031.

### Fixation durations

In the encoding phase, fixation durations were marginally shorter for correctly remembered images (Hits) compared to those that were not (Misses), χ^2^(1) = 3.60, p = 0.058, Odds Ratio = 0.83, CI = [0.69, 1.00], z = -1.99, p = 0.047. In the recognition phase, fixation durations were significantly longer for old images that were correctly recognized (Hits) compared to those that were not (Misses), χ^2^(1) = 36.83, p < 0.001, Odds Ratio = 3.05, CI = [1.97, 4.71], z = 5.03, p < 0.001. Similarly, for new images, fixation durations were significantly longer for correct responses (Correct Rejections) compared to incorrect responses (False Alarms), χ^2^(1) = 4.75, p = 0.029, Odds Ratio = 1.81, CI = [1.04, 3.16], z = 2.08, p = 0.037. Note that there is an inverse relationship between number of fixations and fixation duration, which follows naturally from the fixed, 10-seconds stimulus duration: The more fixations are made, the shorter the fixation durations must be, and vice-versa. The effects of the encoding and recognition phases on the duration of fixations support earlier findings showing that fixation duration is a good indicator of attentional processing [39, 46].

### Exploration tendency of eyes

In the encoding phase, the eyes were more exploratory in images that were correctly recognized (Hits) compared to those that were not (Misses), χ^2^(1) = 7.37, p = 0.007, Odds Ratio = 1.33, CI = [1.08, 1.63], z = 2.74, p = 0.006. Note that due to singularity issues arising from near-zero variance for the random effect of subject, it was dropped from the model [45]. In the recognition phase, the eyes were less exploratory for old images that were correctly recognized (Hits) compared to those that were not (Misses), χ^2^(1) = 53.60, p < 0.001, Odds Ratio = 0.38, CI = [0.28, 0.51], z = -6.54, p < 0.001. For new images, fixations were less exploratory for correct responses (Correct Rejections) compared to incorrect responses (False Alarms), χ^2^(1) = 6.24, p = 0.012, Odds Ratio = 0.97, CI = [0.94, 0.99], z = -2.51, p = 0.012.

### Exploration tendency of head

In the encoding phase, head positions were more exploratory in images that were later correctly recognized (Hits) compared to those that were not (Misses), χ^2^(1) = 3.98, p = 0.046, Odds Ratio = 1.04, CI = [1.00, 1.08], z = 2.00, p = 0.046. In the recognition phase, head positions were less exploratory for old images that were correctly recognized (Hits) compared to those that were not (Misses), χ^2^(1) = 341.14, p < 0.001, Odds Ratio = 0.71, CI = [0.69, 0.74], z = -18.55, p < 0.001. For new images, head positions were less exploratory for correct responses (Correct Rejections) compared to incorrect responses (False Alarms), χ^2^(1) = 18.07, p < 0.001, Odds Ratio = 0.88, CI = [0.83, 0.93], z = -4.26, p < 0.001.

In summary, the extent to which individuals move their eyes and head to explore panoramic scenes is associated with their memory performance. During encoding, more fixations, and more exploration (with both the eyes and head) were associated with scenes that were later correctly recognized. During recognition, correctly recognized images were associated with fewer fixations, but longer fixation durations and less exploration with the eyes and head. There was a similar pattern for correct rejections compared to false alarms, but the magnitude of these differences is less pronounced (see Table 3).

### Spatio-temporal analysis

The spatio-temporal analysis of eyes and head relies on cross-recurrence analysis (CRA; [47, 48]), which is a generalization of recurrence quantification analysis [49]. This technique permits an analysis of eye and head patterns over space and time. CRA allows for the quantification of behavioral similarity among people and images. That is, do certain aspects of eye and head movement behaviour overlap across images, or across people when encoding and recognizing a scene? In this section, we begin with a brief description of cross-recurrence and its most important measures, where each measure quantifies an interesting behavioural (scan or head path) similarity. Then we present an analysis designed to tease out the eye and head movement behaviours that are unique to the individual when viewing panoramic scenes, versus those that are unique to particular scenes.

**Fig 5:**
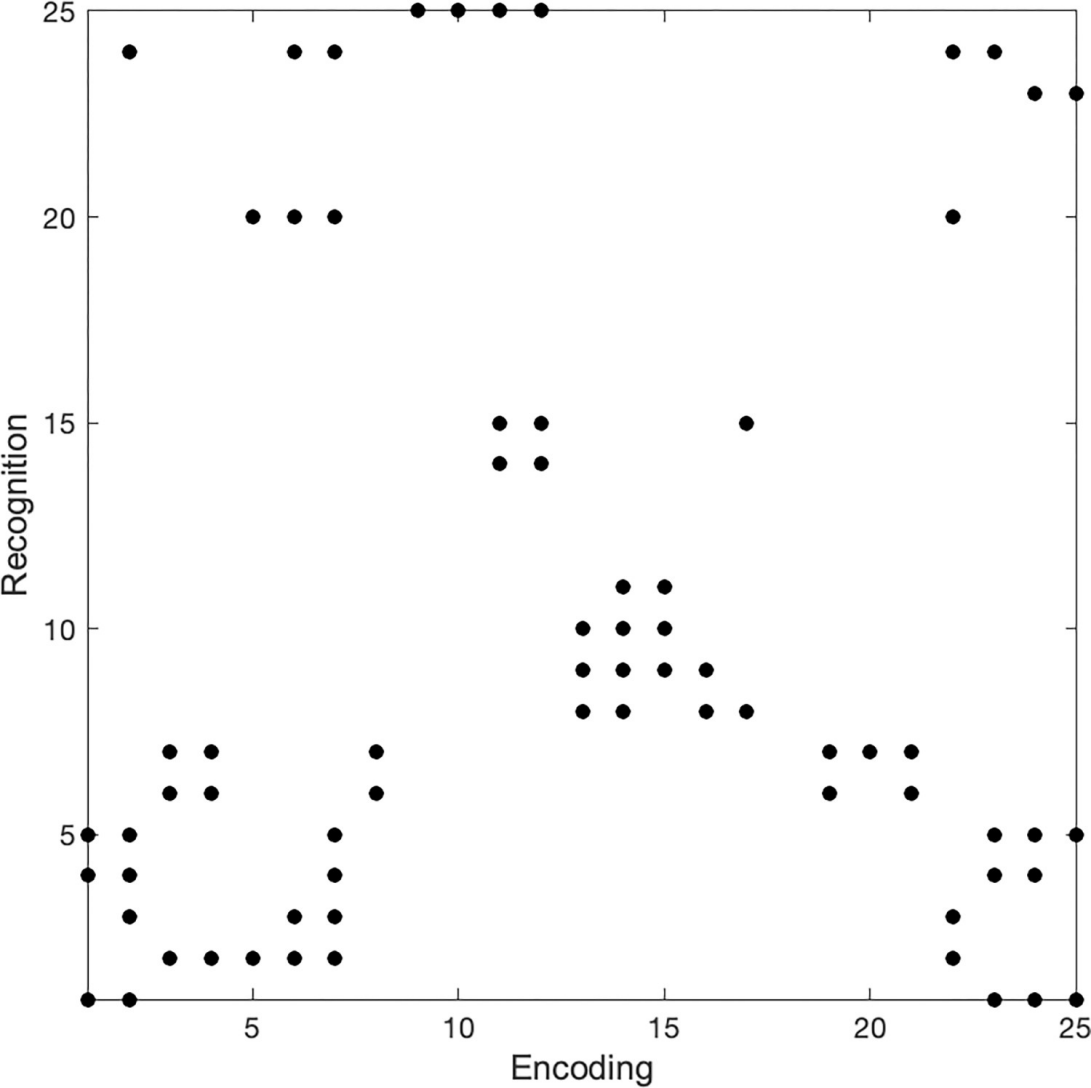
Example of a cross-recurrence diagram. The encoding and recognition fixations are adjusted to 25 fixations, with dots indicating cross-recurrences. For example, encoding fixation 3 and recognition fixations 2, and 6–7, are cross-recurrent, i.e., their angular distance is below the defined recurrence threshold.

Consider two fixation sequences E (for encoding) and R (for recognition), which are adjusted to have the same lengths. Within these two sequences, two fixations e_i_ and r_j_ are cross-recurrent if their angular distance is below a given threshold (see the complete presentation and formal definitions given in S2 Appendix). CRA was computed on the fixation sequences (scan paths) and head position sequences (head paths) for each participant during encoding and recognition. An example of a cross-recurrence diagram is shown in Fig 5.

### Recurrence radius

**Fig 6:**
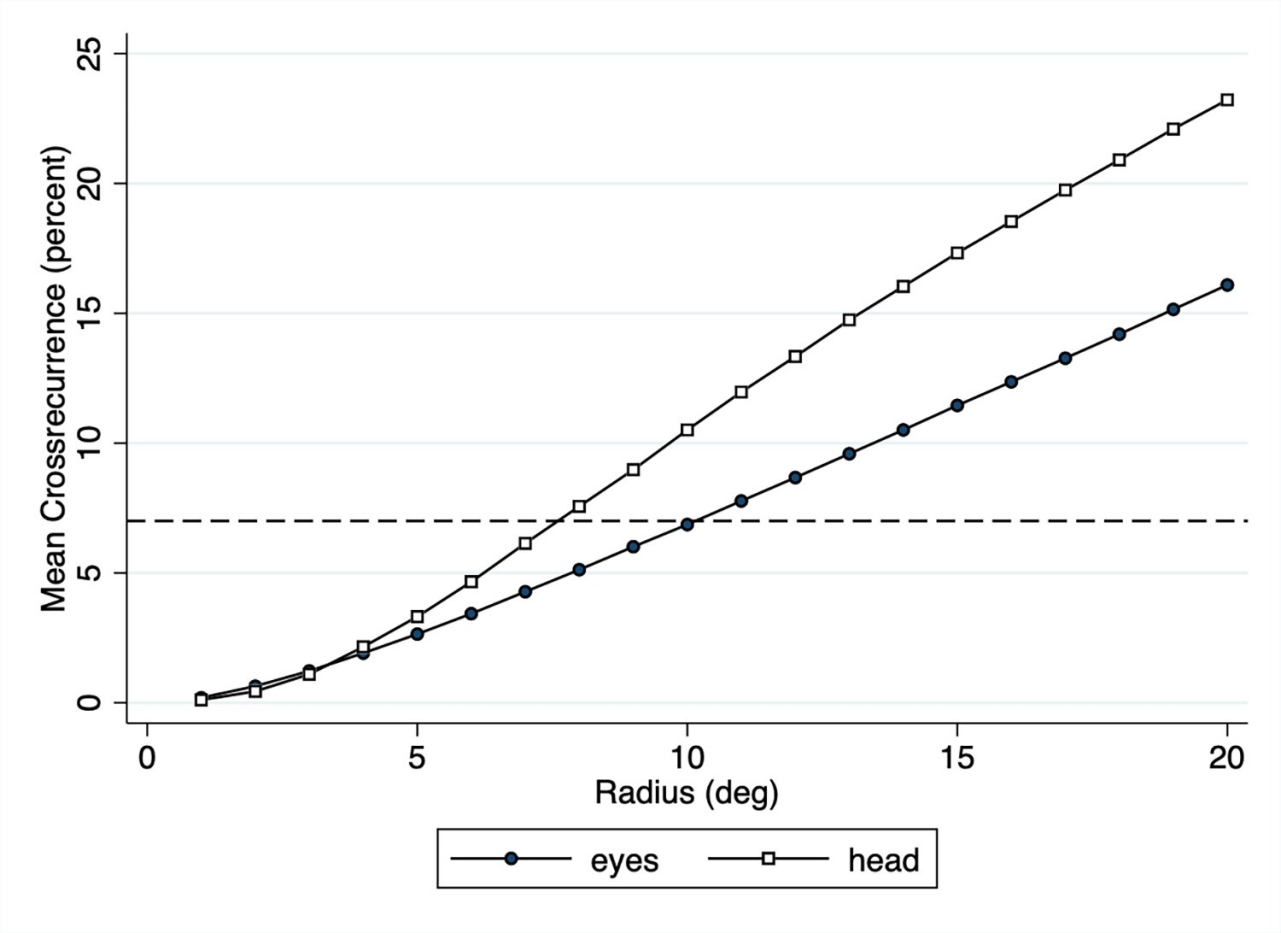
Mean cross-recurrence of eyes and head as a function of cross-recurrence radius. The cross-recurrences were computed for all panoramas used in the experiment. As the radius increases, so does the mean cross-recurrence. We chose a radius of 10.1° for fixations and 7.6° for average head positions to obtain an average cross-recurrence of 7 percent (dashed line).

The relation between encoding and recognition scanpaths were analyzed using cross-recurrence analysis. The CRA measures depend on the recurrence radius, i.e., the average cross-recurrence increases with the radius, which in this case is defined by the angular distance threshold. This is illustrated in Fig 6. As the radius increases, so does the mean cross-recurrence. Given the larger spread of eye points (see Table 1) compared to head points (see Table 2), the two curves diverge as radius increases. Webber and Zbilut [50] suggest several guidelines for selecting the proper radius, including the selection of a radius such that percentage of recurrences remains low. In the present study eye and head patterns could be well characterized using an average cross-recurrence of 7 percent, which is close to the usual percentage of 5 percent while at the same time ensuring that there are sufficiently many recurrences. To obtain an average cross-recurrence of 7 percent for eyes and head (dashed line in Fig 6), we chose a cross-recurrence radius of 10.1° for eye fixations and of 7.6° for head positions.

### CRA measures

Several measures are used to quantify aspects of scanpath similarity through cross-recurrence. The CRA measures below are described in terms of fixations, but the same measures can also be applied to averaged head positions.

- Recurrence (REC) is the percentage of cross-recurrent points (out of the total possible cross-recurrences) and indicates the amount of spatial overlap between encoding and recognition fixations. For example, if the spatial overlap between encoding and recognition fixations for scene 1 is high, the percentage of recurrent points will be high.
- Determinism (DET) is the percentage of these recurrent points that form diagonal lines in the cross-recurrence diagram. This quantifies the number of fixations that overlap in both space and time. A high percentage of deterministic fixations means that there are fixation trajectories that are common to both fixation sequences. For example, sequences of fixations made during encoding that are then exactly repeated during the recognition of a scene.
- Vertical Laminarity (vLAM) and the related Vertical Trapping Time (vTT) quantify the percentage of recurrent points that form vertical lines in the cross-recurrence diagram, with vTT indicating the average length of these vertical lines. This captures the behaviour where an individual briefly scans a particular region during encoding and then inspects it in detail during recognition (see the example in Fig 5 for such an instance).
- Horizontal Laminarity (hLAM) and the related Horizontal Trapping Time (hTT) quantify the percentage of recurrent points that form horizontal lines in the cross-recurrence diagram, with hTT indicating the average length of these horizontal lines. This captures the behaviour where an individual scans a region in detail during encoding and then only briefly inspects it during recognition.
- The Center of Recurrence Mass (CORM) measure quantifies where on the cross recurrence plot the majority of recurrences are. A large positive value indicates that during recognition, observers spend more time at locations looked at earlier on during encoding. A large negative CORM value indicates that during recognition, observers spend more time at locations looked at later during encoding. This measure essentially quantifies primacy (positive CORM) and recency (negative CORM) effects.
- The Clusters (CLUST) measure quantifies the percentage of recurrent fixations that form clusters with at least 8 cross-recurrences in the cross-recurrence diagram. The presence of clusters in the cross-recurrence diagram expresses the tendency to focus on the same spatio-temporal subsets of locations during encoding and recognition.
- The Entropy (ENT) is defined as the entropy of the probability distribution of diagonal lengths. In general, it reflects the complexity of the deterministic structure in a system, in our case of eye or head patterns. For uncorrelated patterns, the value of ENT is small, indicating its low complexity [51].

### Scanpath similarity between encoding and recognition

In the analysis of the CRA data, we were interested in how the two dimensions, Participants and Images, affected the similarity of the encoding and recognition scanpaths as measured by the nine different CRA measures introduced earlier. Same-participant similarity suggests that participant is a factor in the scanpath similarity, and Same-images similarity suggests that the images themselves affect scanpath similarity. The comparisons were made as follows. During encoding, 80 images were shown, with 40 images being shown again during recognition (referred to as the repeated R-set), and 40 images not being shown during Recognition (referred to as the dropped D-set). During recognition, 80 images were shown, 40 Old images that were shown during encoding and 40 New images. To separate the effects of Images and Participant, the following comparisons were made:

- Same-participant Same-images (Sp-Si): For each ‘Old’ recognition trial, the recognition image was paired with the matching image from the R-set.
- Same-participant Different-images (Sp-Di): For each ‘New’ recognition trial, the recognition image was paired with a randomly chosen image from the D-set.
- Different-participant Same-images (Dp-Si): For each ‘Old’ recognition trial, the recognition image was paired with the matching image from the R-set of a randomly chosen other person.
- Different-participant Different-images (Dp-Di): For each ‘New’ recognition trial, the recognition image was paired with a randomly chosen image from the D-set of a randomly chosen other person.

**Fig 7:**
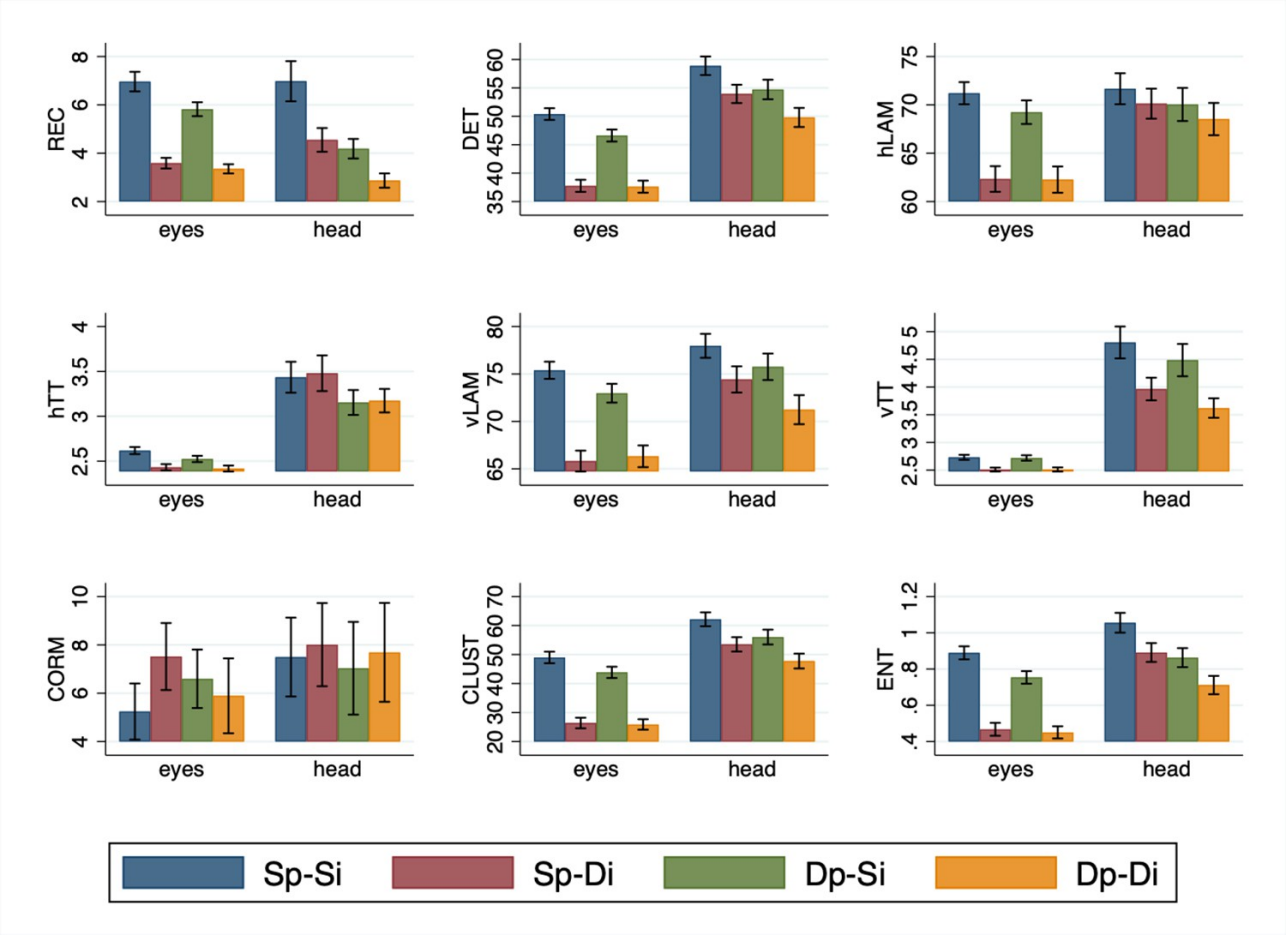
Scanpath similarities for eyes and head measured using nine CRA measures. Scanpath similarity values for eyes and head are shown for the four conditions (Sp-Si, Sp-Di, Dp-Si, Dp-Di) and the nine CRA measures (REC, DET, hLAM, hTT, vLAM, vTT, CORM, CLUST and ENT).

Fig 7 shows the mean scanpath similarities for eyes and head, for nine CRA measures (REC, DET, hLAM, hTT, vLAM, vTT, CORM, CLUST and ENT), across the four conditions (Sp-Si, Sp-Di, Dp-Si and Dp-Di).

An analysis of the eyes results in Fig 7 shows that all measures except CORM showed the same general pattern, namely a strong effect of images and a weak effect of participant. The CRA measures were highest when the same participants looked at the same images during encoding and recognition (Sp-Si). This fixation overlap between encoding and recognition tends to show little or no decline when the encoding and recognition paths of different participants viewing the same images are compared (Dp-Si). This indicates that the contribution of the participant to scanpath overlap is weak, and suggests that the information in the image is driving much of the variance in eye and head movement behaviour. This inference is supported by our finding that in relation to Sp-Si fixation overlap declines profoundly, and generally by the same magnitude, when the same or different participants look at different images (Sp-Di and Dp-Di). Collectively, these data indicate that scanpath overlap is high when the images are the same, and it is low when the images are different. In light of this strong effect of image, if any effect of participant is expressed, it tends to be seen when the images are the same.

These observations are supported by a statistical analysis (see S3 Appendix). We performed a 2 (Participant; Same-Participant vs. Different-Participant) by 2 (Image; Same-Image vs. Different-Image) repeated measures analysis of variance for each CRA measure of the eyes. A significant main effect of Image was return for all measures (F’s > 55.25, p’s < 0.001) except CORM (F = 0.89, p = 0.36). In contrast there was a main effect of Participant only for DET, vLAM, and ENT (F’s > 5.64, p’s < 0.05). Reflecting the fact that any effect of Participant tends to be evident when the images are the same, an Image x Participant interaction was returned for all measures (F’s > 5.67, p’s < 0.05) save for hLAM, vTT and CORM (F’s < 2.90, p’s > .10).

For the head, the pattern of results in Fig 7 is different from that of the eyes. Most CRA measures were again highest when the the head of the same participants are directed at the same images during encoding and recognition (Sp-Si), but the effect is less consistent for the other conditions. This is again supported by a statistical analysis (see S3 Appendix). We performed a 2 (Participant; Same-Participant vs. Different-Participant) by 2 (Image; Same-Image vs. Different-Image) repeated measures analysis of variance for each CRA measure of the head. A significant main effect of Image was returned for all measures (F’s > 19.75, p’s < .001), save for hTT and CORM (F < 0.60, p’s > .45), and brushing significance for hLAM (F = 4.08, p = 0.057). The effect of Participant is present only for vLAM, vTT, and ENT (F’s > 5.90, p’s < 0.05). Consistent with the generally weaker effects for head, no interactions were observed (all F’s < 3.65, p’s > 0.05).

Taken together, the results reveal an interesting relationship between eyes and head. A purely spatial analysis has shown that the eyes stay fairly close to the head-defined center of field of view, supporting earlier results in the literature (e.g., [39, 40]). In contrast, the spatio-temporal analysis shows differential influences of Image and Participant on their relation, with a consistent and strong effect of Image and a weaker effect of Participant on the scanpath similarities of the eyes, but less consistent effects on the scanpath similarities of the head. In other words, the eyes seem to be strongly driven by the image content, and while this is also true for head movements, there seems to be room for more variance arising from the person doing the moving. This agrees with earlier work in VR [27] where there are strong individual differences in head movement propensity (i.e., ‘movers’ and ‘nonmovers’ [52]), and suggests that the eyes and head may be under different control mechanisms.

## Discussion

The present paper addresses three core questions: How does the movement of the head and eyes support the acquisition of panoramic visual information when observers are immersed within VR? How is this movement associated with subsequent scene recognition? And to what extent are similarities in head and eye movements between scene encoding and recognition attributable to general patterns of scene exploration versus exploration patterns that are responsive to the content of the environment itself? To answer these questions, we focused on the spatial and temporal similarities of eye and head movements during the encoding and the recognition of panoramic scenes in VR.

### Spatial analysis

The spatial analysis of eye movements revealed several results. First, the analysis showed a strong horizontal bias of eye fixations, suggesting that participants may have focused primarily on the areas of the panoramic scenes that were most informative [27, 30]. Second, the spread of eye movements was larger in the encoding phase than in the recognition phase, consistent with the notion that participants focus on the exploration of the scenes during the encoding phase, whereas during the recognition phase the emphasis is on the comparison of scene images with stored representations acquired during encoding. Third, the correlational analysis of the encoding and recognition heatmaps showed a very close similarity of the eye movement patterns in the two phases, providing further evidence that in the recognition phase, eye fixations were concentrated on those areas of the panoramic scenes that allowed an informed comparison with those visual representations stored in memory. The analysis of the head data revealed a similar pattern of results, although smaller in absolute terms. Indeed, we found that eye and head movements were themselves more correlated when looking at the same rather than different visual scenes.

### Memory performance

Given that eye and head movements are similar, especially when looking at the same scenes, the question then is whether these similarities are associated with correct memory performance? They are, unequivocally. More exploration of visual scenes during encoding is linked to successful recognition of those scenes later. All our measures converged on this conclusion. Greater spatial exploration with the eyes and head during encoding as well as more and briefer fixations are all associated with enhanced scene recognition. And when those scenes are correctly recognised, observers are more inclined to explore them less with their head and eyes while making fewer and longer fixations relative to those scenes that are not recognised. In sum, both head and eye measures serve as reliable indicators of successful encoding and recognition of panoramic scenes.

### Spatio-temporal analysis

What we still do not know, however, is whether these similarities above reflect general patterns of visual exploration (i.e., those associated with an observer’s idiosyncratic tendencies) versus a sensitivity to the visual environment. To address this issue we conducted a spatio-temporal analysis of eye and head movements using cross-recurrence analysis. For eye movements a consistent pattern of results was found for all CRA measures—except center of recurrence mass (CORM) which measures whether there is a tendency for most recurrences to occur earlier or later in the sequence. Specifically, the analyses revealed that recurrences were higher for the same participants viewing the same images (Sp-Si) than the same participants viewing different images (Sp-Di) or different participants viewing the same images (Dp-Si; Fig 7). The differences between Sp-Si and Sp-Di indicate that changing the image leads to a strong reduction in scanpath similarity (see also Dp-Si versus Dp-Di), and the differences between Sp-Si and Dp-Si indicate that changing the participant leads to a weaker reduction in scanpath similarity (see also Sp-Di versus Dp-Di). Together, these results indicate that the spatio-temporal eye movement patterns are determined primarily by the content of the panoramic scenes and to a lesser degree by patterns of movement particular to the participants themselves.

The spatio-temporal analysis of head positions using cross-recurrence analysis showed a slightly different pattern of effects than for the eyes. Image content was again responsible for the similarity in head path movements, although this effect was relatively weaker than for the eyes. Previous correlational analyses and our spatial analyses of eyes and head show strong similarities, but the spatio-temporal analyses indicate more subtle differences between the two effectors. We consider this relation in more detail below.

### Eyes and head

By immersing individuals in 360° virtual scenes and allowing them to be explored freely enables us to extend the results of the many eye movement laboratory studies that have presented stimuli on computer monitors and discouraged any movement of the head. Moreover, and in contrast to naturalistic experiments outside the laboratory [12, 15, 53], the analysis of our virtual visual environment has the advantage of being unaffected by any number of additional variables such as planning of navigating routes through the environment and obstacle avoidance. This leads to a unique opportunity for studying the relation between the movement of the head and eyes while observers explore, encode, and recognize immersive visual environments.

The head and eyes can work in a similar, coordinated manner without well-defined separate roles in visual exploration [8]. Along these lines, it has been shown, for example, that visual information is encoded more fully, and is better remembered, when it is accompanied by a head movement, presumably because the observer expends more energy when making a head and eye movement [9, 21]. Furthermore, the head and eyes may complement each other where, for example, the head can be used to extend the field of view, altering what the eyes ultimately examine [15, 54–56]. When the periphery is masked using head- or eye- contingent windows, it has been shown that the head is more likely to move into unseen space than the eyes [23], and when tested in VR, it has been shown that head movements are impacted much less by peripheral or central vision loss than eye movements [29]. In this context, head movements are controlled in a top-down, task-oriented manner, enabling a changing field of view; whereas eye movements are controlled in both a top-down and a bottom-up manner to change the focus of attention within the field of view [25, 27, 41, 57]. In the present study we find that the head and eyes are linked, though the effect of image content—which is primarily responsible for an overlap in head- and eye-path movements—is most pronounced for the eyes. This is consistent with the eyes ultimately being responsible for taking in visual information, with head movements supporting that visual acquisition by the eyes.

## Conclusions

The present study harnessed to the power of virtual reality to examine how unconstrained observers use their head and eyes to encode and recognise panoramic visual scenes. By studying head and eye movement within a fully immersive environment, and applying cross-recurrence analysis, we have found that eye movements are strongly influenced by the content of the visual environment, as are head movements—though to a much lesser degree. Moreover, we find that the head and eyes are linked, with the head supporting, and by and large mirroring the movements of the eyes, consistent with the notion that the head operates to support the acquisition of visual information by the eyes.

One of the many strengths of using VR to measure head and eye movements is the sheer wealth of data that one collects [35]. The analyses in the present study were applied to address our research questions but we hasten to add that there are very many questions and analyses that remain to be considered in the future. For instance, we currently do not know what factors are critical for whether a head movement accompanies an eye movement. The distance and the direction that the eyes move is an obvious consideration [25, 38], but as suggested by the present study, the role of the individual may also be a key consideration [52]. Future research questions such as these will also demand other analyses and measures, such as the position of the eyes within the head, their movement direction, the temporal relationship between eye ampliude and the initiation of a head movement, and of course, their relationship to individual difference measures. We are excited by the many new discoveries that remain to be made.

## Supporting information

S1 Appendix Heatmap correlation. (DOCX)

S2 Appendix Cross-recurrence analysis [49, 51, 58]. (DOCX)

S3 Appendix Statistical analysis of CRA measures. (DOCX)
