# Supplementary material for: Eye and head movements while encoding and recognizing panoramic scenes in virtual reality
Source: PLoS One. 2023 Feb 17;18(2):e0282030. doi: 10.1371/journal.pone.0282030 (PMC9937482; doi:10.1371/journal.pone.0282030)
Supplement: S1 Appendix — (DOCX) [file pone.0282030.s001.docx]

**S1 Appendix. Heatmap correlation.**

To compute the correlation between two heatmaps $E_{\mathrm{xy}}$ and $R_{\mathrm{xy}}$ with x∈[-180, 180] and y∈[-90, 90], one must compensate the distortion near the south and north poles, i.e., for y close to -90 and +90. To this purpose, a weight function w_y_ ∝ cos(y) is introduced, and the weighted heatmap correlation r_ER_ is defined as follows.

$$w_{y}=\cos\left( y \right)/\sum_{x=-180}^{180} \sum_{y=-90}^{90} cos(y)$$

$$m_{E}=\sum_{x=-180}^{180} \sum_{y=-90}^{90} w_{y}E_{\mathrm{xy}}$$

$$m_{R}=\sum_{x=-180}^{180} \sum_{y=-90}^{90} w_{y}R_{\mathrm{xy}}$$

$$S_{\mathrm{EE}}=\sum_{x=-180}^{180} \sum_{y=-90}^{90} w_{y}{{(E}_{\mathrm{xy}}-m_{E})}^{2}$$

$$S_{\mathrm{RR}}=\sum_{x=-180}^{180} \sum_{y=-90}^{90} w_{y}{{(R}_{\mathrm{xy}}-m_{R})}^{2}$$

$$S_{\mathrm{ER}}=\sum_{x=-180}^{180} \sum_{y=-90}^{90} w_{y}{(E}_{\mathrm{xy}}-m_{E}){(R}_{\mathrm{xy}}-m_{R})$$

$$r_{\mathrm{ER}}= \frac{S_{\mathrm{ER}}}{\sqrt{S_{\mathrm{EE}}S_{\mathrm{RR}}}}$$

Given N images, with recognition heatmaps $R_{\mathrm{xy}}^{i},$encoding heatmaps $E_{\mathrm{xy}}^{j}$, and the heatmap correlations $r_{\mathrm{ER}}^{\mathrm{ij}}$, let

$$z_{\mathrm{ij}}=\mathrm{atanh} (r_{\mathrm{ER}}^{\mathrm{ij}}),$$

i.e., z_ij_ are the Fisher z-transformations. The hypothesis in the main text states that, for all images, z_ii_ for matching encoding and recognition images is larger than M_i_,

$$M_{i}=\frac{1}{N-1}\sum_{j=1 j\neq i}^{N} z_{\mathrm{ij}},$$

the average for the non-matching images.
