# Supplementary material for: Eye and head movements while encoding and recognizing panoramic scenes in virtual reality
Source: PLoS One. 2023 Feb 17;18(2):e0282030. doi: 10.1371/journal.pone.0282030 (PMC9937482; doi:10.1371/journal.pone.0282030)
Supplement: S2 Appendix — (DOCX) [file pone.0282030.s002.docx]

**S2 Appendix. Cross-recurrence analysis.**

Consider two fixation sequences **E** = **e**_i_, i=1, …, N, with **e**_i_ = <x_i_,y_i_> and **R** = **r**_j_, j=1, …, N, with **r**_j_ = <x_j_,y_j_>. For fixation sequences of unequal length, the longer sequence is truncated. Two fixations **e**_i_ and **r**_j_ are cross-recurrent if they are close together, i.e., we define the cross-recurrence of two fixations c_ij_ as

|  | $c_{\mathrm{ij}}=\left\{ \begin{aligned} 1, &d(\mathbf{e}_{\mathbf{i}}, \mathbf{r}_{\mathbf{j}})\leq\rho\\ 0, &\mathrm{otherwise} \end{aligned} \right.$ |
| --- | --- |

where d is a distance measure (e.g., Euclidian distance in 2D or the great circle distance in spherical displays) and ρ is a threshold.

Several measures can be used for characterizing cross-recurrence patterns. The measures are extensions of the recurrence measures introduced by Anderson et al. [49]. In the cross-recurrence matrix **C** = c_ij_, i=1, …, N, j=1, …, N; let D be a diagonal line representing a fixation trajectory common to both fixation sequences; let L_H_ be a horizontal laminate line, representing a single fixation in **r** being recurrent with a sequence of fixations in **e**; and let L_V_ be vertical laminate line, representing a single fixation in **e** being recurrent with a sequence of fixations in **r**. D, L_H_ and L_V_ are taken into account only if their lengths are at least L_min_ (usually L_min_=2). Finally, let C be the sum of recurrences, i.e., $C=\sum_{i=1}^{N} \sum_{j=1}^{N} c_{\mathrm{ij}}$ and let |·| denote cardinality.

The Cross-Recurrence measure of two fixation sequences is defined as

$$REC=100\cdot\frac{C}{N^{2}}.$$

It represents the percentage of cross-recurrent fixations, i.e., the percentage of fixations that match (i.e., are close) between the two fixation sequences.

The Determinism measure is defined as

$$DET=100 \cdot\frac{\left| D \right|}{C}.$$

It is the percentage of cross-recurrent points that form diagonal lines and represents the percentage of fixation trajectories common to both fixation sequences. That is, it quantifies the overlap of a specific sequence of fixations, preserving the sequential information.

The vertical laminarity measure is defined as

$$\text{vLAM}= 100\cdot\frac{|L_{V}|}{C}.$$

It is the proportion of fixations in vertical laminate lines L_V_ and represents the percentage of fixation that were fixated in detail in **r**, but only fixated briefly in **e**.

The horizontal laminarity measure is defined as

$$\text{hLAM}= 100\cdot\frac{|L_{H}|}{C}.$$

It is the proportion of fixations in horizontal laminate lines L_H_ and represents the percentage of fixation that were fixated in detail in **e**, but only fixated briefly in **r**.

The vertical trapping time vTT is defined as the average length of all vertical laminate lines L_V_.

The horizontal trapping time hTT is defined as the average length of all horizontal laminate lines L_H_.

A cluster in the cross-recurrence matrix is defined by the following connectivity criterion: If the cross-recurrence c is in a cluster, then the cross-recurrences in the 8-connected region of c are also in the cluster [58]. Only clusters with at least 8 cross-recurrences are counted. The CLUST measure is defined as the percentage of cross-recurrent fixations that are part of a cluster.

The center of recurrence mass CORM is defined as the distance of the center of gravity from the main diagonal, normalized such that the maximum possible value is 100,

| $CORM=100\frac{\sum_{i=1}^{N} \sum_{j=1}^{N} \left( j-i \right)r_{\mathrm{ij}}}{(N-1)C}$. |  |
| --- | --- |

The CORM measure indicates the dominant lag of cross-recurrences. Small corm values indicate that the same fixations in both fixation sequences tend to occur close in time, whereas large corm values indicate that cross-recurrences tend to occur with either a large positive or negative lag. CORM values are positive if the fixation sequence **e** is leading the fixation sequence **r**, and it is negative if the fixation sequence **r** is leading the fixation sequence **e**.

The cross-recurrence entropy ENT is defined as the entropy of the probability distribution of diagonal line lengths p(D),

$ENT=-\sum_{l=l_{\min}}^{N} p\left( D \right)\text{ln}p(D)$.

ENT reflects the complexity of the deterministic structure in a system, in our case of gaze or head patterns. For uncorrelated patterns, the value of ENT is small, indicating a low complexity [51].
