# Supplementary material for: Eye and head movements while encoding and recognizing panoramic scenes in virtual reality
Source: PLoS One. 2023 Feb 17;18(2):e0282030. doi: 10.1371/journal.pone.0282030 (PMC9937482; doi:10.1371/journal.pone.0282030)
Supplement: S3 Appendix — (DOCX) [file pone.0282030.s003.docx]

**S3 Appendix. Statistical analysis of CRA measures.**

Gaze data were analyzed using a 2 (Participant; within-Participants vs. between-Participants) by 2 (Image; within-Images vs. between-Images) repeated measures analysis of variance for each scanpath measure. Table C1 presents, for each scanpath measure, the F-ratio, p-values and effect size ${}_{p}^{2}$ for the main effect of Participant, the main effect of Image, and the Participant-by-Image interaction.

**Table S3-1. Anova of CRA measures for eyes data.**

| Measure | Participant | | | Image | | | Participant x Image | | |
| --- | --- | --- | --- | --- | --- | --- | --- | --- | --- |
|  | F | p | ${}_{p}^{2}$ | F | p | ${}_{p}^{2}$ | F | p | ${}_{p}^{2}$ |
| REC | 3.04 | .097 | 0.132 | 131.21 | **< .001** | 0.868 | 9.21 | **.007** | 0.315 |
| DET | 10.28 | **.004** | 0.339 | 731.43 | **< .001** | 0.973 | 28.47 | **< .001** | 0.587 |
| hLAM | 0.61 | .445 | 0.030 | 153.14 | **< .001** | 0.884 | 2.86 | .107 | 0.125 |
| hTT | 1.83 | .192 | 0.084 | 55.27 | **< .001** | 0.734 | 5.67 | **.027** | 0.221 |
| vLAM | 5.64 | **.028** | 0.220 | 168.46 | **< .001** | 0.894 | 19.83 | **< .001** | 0.498 |
| vTT | 0.01 | .909 | 0.001 | 69.47 | **< .001** | 0.776 | 0.49 | .492 | 0.024 |
| CORM | 0.03 | .870 | 0.001 | 0.89 | .357 | 0.043 | 4.39 | .049 | 0.180 |
| CLUST | 3.00 | .099 | 0.130 | 409.67 | **< .001** | 0.953 | 12.68 | **.002** | 0.388 |
| ENT | 9.51 | **.006** | 0.322 | 310.97 | **< .001** | 0.940 | 23.10 | **< .001** | 0.536 |

The table gives F-ratios, p-values and ${}_{p}^{2}$ for each CRA measure, for the main factors Participant and Image and the interaction Participant x Image. All significant effects are bolded.

Head data were also analyzed using a 2 (Participant; within-Participants vs. between-Participants) by 2 (Image; within-Images vs. between-Images) repeated measures analysis of variance for each scanpath measure. Table C2 presents, for each scanpath measure, the F-ratio, p-values and effect size ${}_{p}^{2}$ for the main effect of Participant, the main effect of Image, and the Participant-by-Image interaction.

**Table S3-2. Anova of CRA measures for head data.**

| Measure | Participant | | | Image | | | Participant x Image | | |
| --- | --- | --- | --- | --- | --- | --- | --- | --- | --- |
|  | F | p | ${}_{p}^{2}$ | F | p | ${}_{p}^{2}$ | F | p | ${}_{p}^{2}$ |
| REC | 3.15 | .091 | 0.136 | 30.85 | **< .001** | 0.607 | 3.61 | .072 | 0.153 |
| DET | 3.93 | .061 | 0.164 | 33.66 | **< .001** | 0.627 | 0.00 | .979 | < 0.001 |
| hLAM | 0.54 | .471 | 0.026 | 4.08 | .057 | 0.170 | 0.01 | .936 | < 0.001 |
| hTT | 1.01 | .327 | 0.048 | 0.03 | .860 | 0.002 | 0.00 | .992 | < 0.001 |
| vLAM | 10.18 | **.005** | 0.337 | 19.78 | **< .001** | 0.497 | 0.87 | .362 | 0.042 |
| vTT | 5.99 | **.024** | 0.230 | 20.12 | **< .001** | 0.502 | 0.12 | .733 | 0.006 |
| CORM | 0.31 | .582 | 0.015 | 0.60 | .447 | 0.029 | 0.01 | .923 | < 0.001 |
| CLUST | 3.52 | .075 | 0.150 | 37.50 | **< .001** | 0.652 | 0.01 | .907 | < 0.001 |
| ENT | 5.91 | **.027** | 0.228 | 57.33 | **< .001** | 0.741 | 0.11 | .739 | 0.006 |

The table gives F-ratios, p-values and ${}_{p}^{2}$ for each CRA measure, for the main factors Participant and Image and the interaction Participant x Image. All significant effects are bolded.
